# Jacalin-Related Lectin *OsJacLK1* Positively Regulates Resistance to *Magnaporthe oryzae* in Rice

**DOI:** 10.3390/plants15091376

**Published:** 2026-04-30

**Authors:** Bingwei Chen, Ruixue Li, Meiling Lai, Haoming Li, Zhongyuan Lin, Sarah Violet Michael, Wenbo Zhu, Jianbo Huang, Songbiao Chen, Yijuan Han

**Affiliations:** 1 Marine and Agricultural Biotechnology Laboratory, Fuzhou Institute of Oceanography, College of Geography and Oceanography, Minjiang University, Fuzhou 350108, China; 2 College of Life Sciences, Fujian Agriculture and Forestry University, Fuzhou 350002, China; 3 College of Plant Protection, Fujian Agriculture and Forestry University, Fuzhou 350002, China

**Keywords:** rice (*Oryza sativa*), jacalin-related lectin kinase, *Magnaporthe oryzae*, disease resistance, defense response

## Abstract

Jacalin-related lectins play crucial roles in plant adaptation to abiotic and biotic stresses. The rice genome encodes four putative jacalin-related lectin kinase genes (*OsJacLKs*), but their functions toward environmental stresses remain largely uncharacterized. This study demonstrates that a putative jacalin-related lectin kinase, *OsJacLK1,* conferred resistance to the rice blast fungus *Magnaporthe oryzae* rather than salt stress. OsJacLK1 protein exhibited agglutination activities and affinity toward chitin, fungal cell wall, and mannose. *OsJacLK1* was transcriptionally activated by stress-related phytohormones salicylic acid (SA), methyl jasmonate (MeJA), abscisic acid (ABA), and indoleacetic acid (IAA), as well as salinity, chitin, and *M. oryzae* inoculation, suggesting its involvement in broad stress-responsive signaling pathways. Overexpression of *OsJacLK1* in rice led to reduced susceptibility to rice blast disease, whereas loss-of-function *osjaclk1* lines showed no significant phenotypic difference from wild-type plants upon infection. Enhanced resistance in *OsJacLK1*-overexpressing lines was associated with a stronger reactive oxygen species (ROS) burst and elevated hydrogen peroxide accumulation, accompanied by the up-regulation of defense-related genes (*OsRac1*, *OsSGT1*, *OsMAPK6*, *OsPAL1*, *OsNAC4*, *OsPBZ1*, *OsAOS2*, and *OsJAZ8*). Collectively, our findings establish that *OsJacLK1* acts as a positive regulator of rice immunity against *M. oryzae*, modulating the cellular redox state, highlighting its potential as a candidate for genetic improvement of disease resistance in rice.

## 1. Introduction

Rice (*Oryza sativa* L.) is the primary staple food crop and a key contributor to global food security [1,2]. Its cultivation is often affected by various environmental stresses, which can significantly impact growth, development, yield, and quality [3,4]. Among these stresses, salinity is one of the most detrimental abiotic factors, severely impairing the growth, development, and productivity of rice and other crops [5,6]. Concurrently, rice blast disease, caused by the filamentous fungus *Magnaporthe oryzae*, poses a major threat to rice production, infecting multiple tissues, including leaves, stems, panicles, seeds, and roots, and resulting in yield losses ranging from 11% to 30% [7,8]. Therefore, understanding the mechanisms underlying both salinity tolerance and resistance to rice blast is crucial for developing resilient rice varieties capable of withstanding stresses.

Lectins are a diverse group of non-enzymatic, carbohydrate-binding proteins or glycoproteins, ubiquitously present in animals, plants, and fungi. These molecules possess a wide range of biological functions, including antiviral, antitumor, antibacterial, antifungal, and insecticidal activities, which are mediated through their ability to specifically recognize and bind carbohydrate structures [9,10]. Jacalin-related lectins (JRLs) are a distinct subgroup within the lectin superfamily, characterized by the presence of one or more jacalin domains [10,11]. They are categorized into galactose-binding and mannose-binding subfamilies, with the prototype jacalin hemagglutinin from *Maclura pomifera,* specifically recognizing Gal-β-(1,3)-GalNAc residues [12], while the sunflower protein Helja binds mannose and inhibits the growth of *Sclerotinia sclerotiorum* by inducing cytotoxicity [13,14]. Beyond carbohydrate recognition and anti-fungal and anti-tumor activities in vitro, jacalin-related lectins are increasingly recognized as crucial modulators of plant growth, development, and stress responses [15]. For instance, silencing the wheat gene *TaJRLL1* results in reduced resistance to *Fusarium graminearum* and rust fungus *Blumeria graminis*, while its heterologous expression in *Arabidopsis* enhances resistance to *F. graminearum* and *Botrytis cinerea* [16]. Similarly, overexpression of the wheat mannose-binding lectin *Ta-JA1* in tobacco confers enhanced resistance to pathogenic bacteria, fungi, and viruses, and also increases barley resistance to *M. oryzae* and *Puccinia hordei* [17,18]. Root-accumulated jacalin, such as *HvHorcH* from barley [19] and *JAL10* from Arabidopsis [20], has been shown to function as a positive regulator of salinity tolerance. Moreover, ectopic expression of the sugarcane dirigent-jacalin gene (*ShDJ*) in rice exhibits improved drought tolerance and plant biomass accumulation [21].

In rice, 30 jacalin-related lectin genes were predicted and catalogued into merojacalins (harboring a single jacalin domain, 16/30), holojacalins (multi-jacalin domain, 1/30), and chimeric jacalins fused with other functional domains (13/30) [22]. Despite their abundance, only a few jacalin genes have been functionally characterized, particularly in relation to stress responses. Notably, *OsJAC1*, a chimeric jacalin containing both a jacalin and a dirigent domain, enhances resistance to multiple pathogens (including *M. oryzae*, *Rhizoctonia solani*, and *Xanthomonas oryzae*) [23,24,25]. Overexpression of *OsJAC1* in Arabidopsis exhibits resistance to ionizing radiation [26]. Another well-studied jacalin, OsMBL1 (also known as SalT or Orysata, JRL), is a multifunctional merojacalin. It contributes to blast resistance by competing for chitin binding with *M. oryzae* chitinase MoChi1 [27] and modulating defense signaling by suppressing several negative regulators in rice [28,29,30]. Rice lines overexpressing *OsMBL1* displayed enhanced tolerance to salt stress [31], but showed reduced tolerance to cold [32]. Furthermore, other JRLs like JRL40 and JRL45 also play a positive role in salt tolerance, underscoring the diverse and critical functions of this family in stress adaptation [30,31,32,33,34,35]. The rice genome encodes four putative jacalin-related lectin kinase (*JacLK*) genes containing a Pkinase domain and at least one jacalin domain. The majority of JacLKs were found in Poaceae species [22,36], suggesting that these genes likely function in specific immune or environmental adaptation mechanisms within the grass family. Nevertheless, their biological functions, especially in stress tolerance, are still unknown. *OsJacLK1* (LOC_Os11g39530), a rice JacLK, was transcriptionally activated at the early stage of *M. orzyae* inoculation via RNA-seq analysis [22], revealing a potential involvement in rice immunity. Therefore, we investigated its responses to stress-related stimuli, including phytohormones, salt stress, and *M. oryzae* infection. To this end, we generated overexpression lines and gene knockout lines, evaluated their contribution to salt stress tolerance and blast resistance. We found that *OsJacLK1* mutants displayed no difference from wild-type ZH11 upon salt stress under the tested conditions; however, the overexpression of OsJacLK1 conferred enhanced resistance against rice blast disease. We further elucidated the underlying molecular mechanisms, including reactive oxygen species (ROS) dynamics and defense gene activation. Our work establishes *OsJacLK1* as a positive regulator of rice immunity against *M. oryzae*, highlighting its potential as a target for genetic improvement of disease resistance.

## 2. Results

### 2.1. Bioinformatic Characterization of OsJacLK1

**Figure 1:**
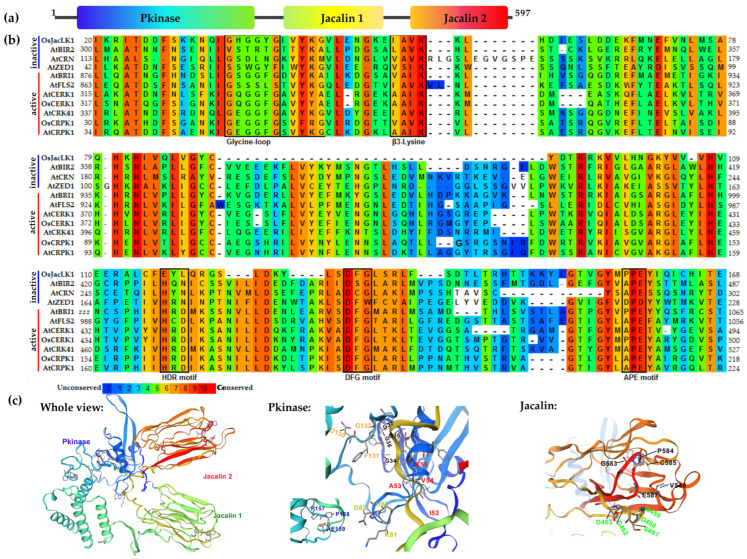
Bioinformatics of OsJacLK1 protein. (**a**) Schematic diagram of the OsJacLK1 protein domain; (**b**) multiple sequence alignment with inactive receptor-like kinases (AtBIR1, AtZED1, AtCORYNE, AtBIR2) and active receptor-like kinase (AtBR1, AtFLS2, AtCRK41, AtCRPK1, AtCERK1, OsCERK1); (**c**) three-dimensional view of OsJacLK1 (SWISS-MODEL). Left, secondary structure of OsJacLK1; middle, ATP-binding sites and catalytic sites in Pkinase domain; right, carbohydrate-binding motif in jacalin domain.

OsJacLK1 encodes a putative jacalin lectin serine/threonine protein kinase with one N-terminal Pkinase domain and two jacalin domains (Figure 1a). The C-terminal region of OsJacLK1 includes a glycan-binding motif GXXXD (e.g., GGSGD), N-glycosylation sites (411NGS, 551NRT), and O-glycosylation sites (such as 251TPS, 270SPS, etc.), indicating that OsJacLK1 is a carbohydrate-binding protein. Additionally, the protein sequence contains numerous phosphorylation sites, such as 138S, 145T, 146T, 152T, 196S, 270S, 433S, 461S, etc. To investigate the phylogeny of OsJacLK1, we constructed a phylogenetic tree by using the amino acid sequence of OsJacLK1 and its plant homologs. JacLKs exhibit a relatively narrow distribution in the plant kingdom, being present mainly in cereal crops (rice, maize, wheat, barley, and sorghum) and a few non-cereal species, such as Rocky Mountain columbine (*Aquilegia coerulea*) and earthmoss (*Physcomitrium patens*) (Figure S1a). OsJacLK1 and its rice paralogs were phylogenetically clustered in the same branch as their homologs in maize and wheat. This grass-specific subclade was clearly separated from the JacLK sequences of columbine and earthmoss.

To evaluate the catalytic potential of JacLK proteins, we performed multiple sequence alignments of JacLK homologs from plants (Figure S1b), as well as canonical inactive/active receptor-like kinases (Figure 1b). Both the N-terminal kinase domain and C-terminal jacalin domain(s) were presented in OsJacLKs and homologs. Compared with inactive/active receptor-like kinases, OsJacLK1 retains several motifs involved in ATP binding and phosphate stabilization [37]: phosphate-anchoring motif GxGxxG (Glycine loop), ATP-anchoring motif IAVK (β3-Lysine), α-C helix glutamate motif, DFG motif, and a PPE motif (Figure 1b,c), all of which were also observed in JacLK homologs. The core catalytic motif of HXD (normally HRD) is absent or substituted in OsJacLK1, whereas it is present in its homologs. It suggests that OsJacLK1 may bind to ATP but likely lacks autophosphorylation activity, and it is unable to catalyze phosphate transfer to substrate proteins.

### 2.2. Characterization of OsJacLK1

**Figure 2:**
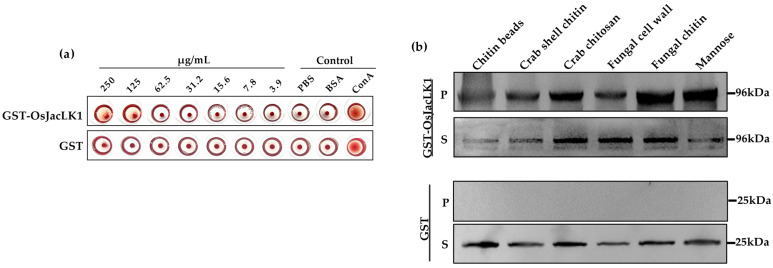
Lectin activity assay and expression pattern of OsJacLK1. (**a**) Agglutinating activity assay of recombinant GST-OsJacLK1. GST, protein buffer PBS, and Bovine Serum Albumin (BSA, 1 mg/mL) were used as a negative control. Concanavalin A lectin (ConA, 11.0 μg/mL) was applied as a positive control; (**b**) Carbohydrate-binding assay of recombinant GST-OsJacLK1. At the top, P represents GST-OsMBL1 co-precipitated with the insoluble polysaccharide chitin magnetic beads, crab shell chitin, chitosan, fungal cell wall from *M. oryzae* (fungal cell wall), chitin from *M. oryzae* (fungal chitin), and mannose; S represents supernatant fractions. At the bottom, for P and S, the mock GST protein was used as a control and only stayed in the supernatants. GST-tagged proteins were detected with an immune blot by using an anti-GST antibody.

In the hemagglutination assay, Concanavalin A (ConA) was used as a positive control and exhibited strong hemagglutination activity, whereas the GST tag protein alone and buffer controls showed no detectable hemagglutination toward rabbit red blood cells. The recombinant OsJacLK1 protein displayed hemagglutination activity with a minimum concentration of 125 μg/mL required for agglutination (Figure 2a).

We also assessed the carbohydrate-binding properties of the OsJacLK1 recombinant protein (Figure 2b) and found that it exhibited affinity for mannose and a variety of chitin forms, including commercial chitin beads, crab shell chitin particles, deacetylated chitin derivative (chitosan), *M. oryzae* cell wall, and *M. oryzae* chitin (Figure 1b, top layer). These results demonstrate that OsJacLK1 is a lectin and binds both chitin and mannose.

### 2.3. OsJacLK1 Was Activated by Stress-Associated Plant Hormone

To explore the potential role of *OsJacLK1* in hormone-mediated defense responses, its transcriptional levels were monitored in wild-type rice plants following treatment with salicylic acid (SA, 1 mM), methyl jasmonate (MeJA, 100 μmol/L), abscisic acid (ABA, 10 μM), and indoleacetic acid (IAA, 20 μM) over various time courses, with water-treated plants serving as the control.

**Figure 3:**
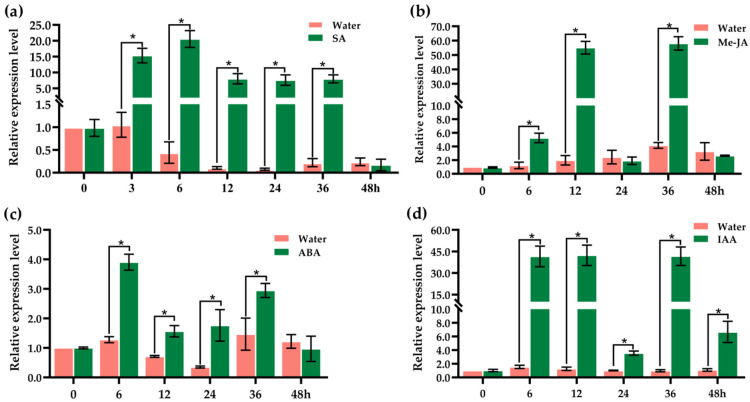
Transcriptional response of *OsJacLK1* to plant hormones. (**a**) Salicylic acid (SA); (**b**) methyl jasmonate (Me-JA); (**c**) abscisic acid (ABA); (**d**) indole-3-acetic acid (IAA). Data are presented as mean ± SD; * represents significant differences at *p* < 0.05 (one-way ANOVA).

Under SA treatment (Figure 3a), *OsJacLK1* expression was strongly induced, increasing sharply to approximately 15-fold at 3 h and peaking at 20-fold at 6 h, compared to consistently low expression observed in the water control. Although expression declined after 6 h, they remained substantially above control levels (7- to 8-fold) up to 36 h. By 48 h, SA-treated expression dropped sharply to 0.17, falling below the control (0.24), suggesting that *OsJacLK1* is activated as an early response to SA.

Upon Me-JA treatment (Figure 3b), *OsJacLK1* transcript levels showed a strong but fluctuating induction pattern. Expression rose 5-fold at 6 h, peaked dramatically to 55-fold at 12 h, then dropped suddenly to the control level at 24 h (1.92 vs. 2.44). A second major peak occurred at 36 h (58.10 vs. 4.17), followed by a decline at 48 h (2.65 vs. 3.28), revealing a potential role for *OsJacLK1* in the early signaling pathway associated with JA-mediated defense responses.

In response to ABA treatment (Figure 3c), *OsJacLK1* expression increased rapidly, reaching a peak of 4.0 at 6 h (vs. 1.28 in the control). Levels remained above control at 12 h and 24 h, rebounded at 36 h (3.0 vs. 1.5), and then declined to near control levels at 48 h (0.97 vs. 1.22), showing that ABA up-regulates *OsJacLK1* expression, particularly at early time points.

Under IAA treatment (Figure 3d), *OsJacLK1* was strongly and rapidly induced. Expression surged to 42-fold at 6 h and remained elevated at 12 h (42.37-fold), followed by a sharp decline at 24 h (3.56-fold). A second induction peak occurred at 36 h (41.73-fold), with a subsequent decrease at 48 h (6.66-fold). These results demonstrate that *OsJacLK1* is highly responsive to IAA, with two distinct induction peaks at 6–12 h and 36 h.

Together, these findings indicate that *OsJacLK1* is broadly responsive to phytohormonal signals. While SA, ABA, and IAA promote sustained induction, Me-JA triggers a dramatic but fluctuating expression.

### 2.4. OsJacLK1 Was Responsive to Salt Stress

**Figure 4:**
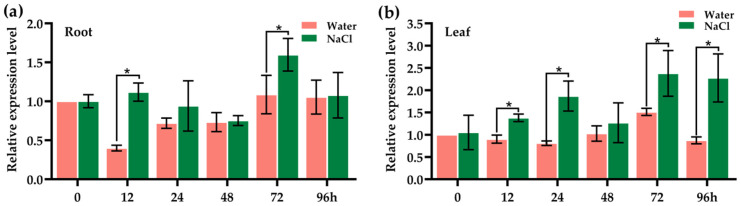
Expression levels of *OsJacLK1* in root and leaf upon salt stress. (**a**) Root; (**b**) leaf. Data are presented as mean ± SD; Data are presented as mean ± SD; * represents significant differences at *p* < 0.05 (one-way ANOVA).

The transcriptional expression of *OsJacLK1* was examined in different tissues (roots, stems, and leaves) of rice cultivars Zhonghua 11 (ZH11), Nipponbare (NPB), and CO39. High expression of *OsJacLK1* was detected in roots, whereas lower levels were observed in the stem and leaves (Figure S2). We then monitored the response patterns of *OsJacLK1* in roots and leaves upon salinity stress. In root, *OsJacLK1* expression was significantly induced at 12 h by salt treatment (NaCl). However, its expression remained largely unaffected after 24 h by salt treatment, except for a modest increase (about 1.5-fold) at 72 h compared with the control (Figure 4a). In leaves, *OsJacLK1* was gradually induced, increasing slightly over time and peaking at 72–96 h (about 2.3-fold) (Figure 4b), indicating that *OsJacLK1* exhibited a modest transcriptional response to salinity stress.

### 2.5. OsJacLK1 Was Activated by Chitin Oligosaccharides and Rice Blast Fungus

**Figure 5:**
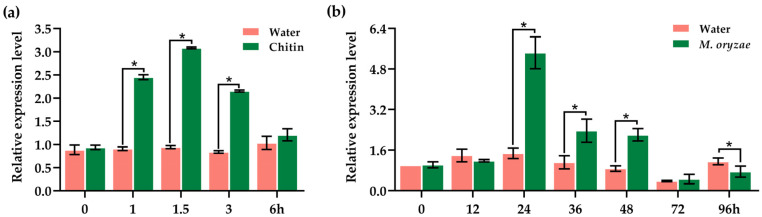
Dynamic change in *OsJacLK1* toward chitin and *M. oryzae*. (**a**) Transcriptional induction of *OsJacLK1* by chitin oligosaccharide (500 μg/mL). (**b**) Transcriptional profiles of *OsJacLK1* in ZH11 by inoculation with *M. oryzae* strain Guy11. Data are presented as the mean ± SD. Asterisks show statistical significance between treatment and water control at the same time point (*p* < 0.05, one-way ANOVA).

Upon treatment with chitin oligosaccharides (Figure 5a), the profiles of *OsJacLK1* gene were rapidly induced, rising to 2.45-fold at 1 h and peaking at 1.5 h (3.08-fold), compared to stable control levels (0.84- to 1.03-fold). The expression gradually declined to 2.15 at 3 h and reached near control levels by 6 h (1.21 vs. 1.03). These results illustrate that *OsJacLK1* is rapidly but transiently up-regulated in response to chitin oligosaccharides at the early stage of signal perception.

Following inoculation with *M. oryzae* (Figure 5b), *OsJacLK1* expression showed a delayed but clear induction. Transcripts of *OsJacLK1* rose to 5.44-fold and peaked at 24 h (vs. 1.48 in control), then decreased but remained above control levels at 36 h (2.37 vs. 1.12) and 48 h (2.21 vs. 0.88). By 72 h and 96 h, expression dropped to near or below control levels. Collectively, these observations indicate that *OsJacLK1* was transcriptionally activated by *M. oryzae* with a pronounced induction at the early stage of infection.

### 2.6. OsJacLK1 May Be Dispensable for Salinity Tolerance in Rice

**Figure 6:**
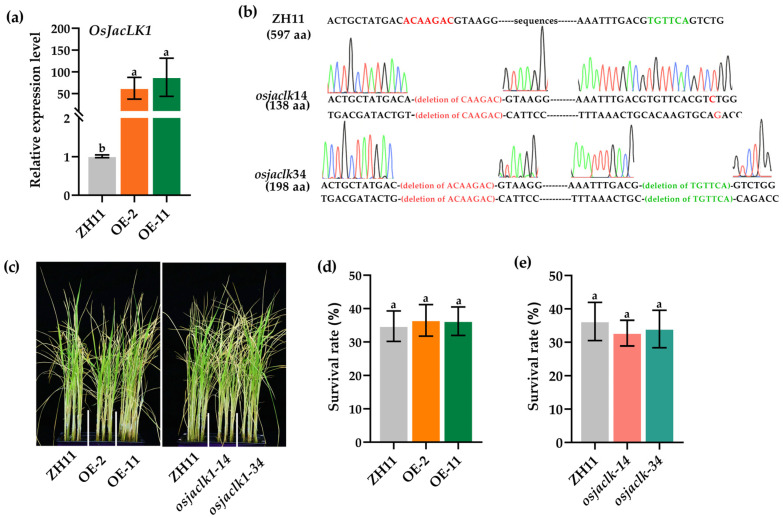
Sensitivity assay of *OsJacLK1* rice mutant plants upon salt stress. (**a**) Transcripts of *OsJacLK1* in *OsJacLK1* overexpression rice mutants. (**b**) Genotypes of CRISPR-Cas9-mediated gene-editing lines *osjaclk1-14* and *osjaclk1-34* by Sanger sequencing. In chromatograms, green represents A (adenine), red represents T (thymine), blue represents C (cytosine), and black (or dark blue) represents G (guanine). (**c**) *OsJacLK1* mutant plants were challenged with NaCl (150 mM) for 7 d. (**d**) The survival rates of *OsJacLK1* overexpression mutant plants under salt stress. (**e**) The survival rates of *OsJacLK1* gene-loss mutant plants under salt stress. Data are presented as the mean ± SD. Different alphabet letters above the columns indicate significant differences between groups (*p* < 0.05, one-way ANOVA followed by Tukey’s post hoc test), whereas groups sharing the same letter are not significantly different.

To investigate the role in stress tolerance, we generated *OsJacLK1* gene-overexpressing lines (OE-2, OE-11) and gene knockout lines (*osjaclk1*-*14* and *osjaclk1*-*34*), all in the ZH11 rice background. The overexpression lines (OE-2 and OE-11) were quantitatively confirmed to exhibit significantly higher expression levels of *OsJacLK1* (>40-fold) compared to wild-type rice (Figure 6a). Homozygous CRISPR/Cas9 editing rice plants (*osjaclk1-14* and *osjaclk1-34*) were verified by sequencing. In *osjaclk1-14* line, two deletions were identified: ACAAGAC at positions 276 to 283 bp and TGTTCA at positions 514 to 520 bp, resulting in a premature stop codon at amino acid position 139. In the *osjaclk1-34* line, a CAAGAC deletion occurred at positions 277 to 283 bp, along with a single cytosine (Cys, C) insertion at position 515 bp, causing a premature termination at amino acid position 199 (Figure 6b).

To assess contributions to salinity tolerance, all *OsJacLK1* mutant lines were subjected to NaCl (150 mM) stress. Although *OsJacLK1* was transcriptionally induced by salinity stress, no obvious and statistical differences were observed among overexpression lines, gene-loss-function lines and wild-type ZH11 under the tested conditions (Figure 6c–e), suggesting that *OsJacLK1* might not play a major role in salt tolerance.

### 2.7. Overexpression of OsJacLK1 Enhanced Rice Resistance Against M. oryzae

**Figure 7:**
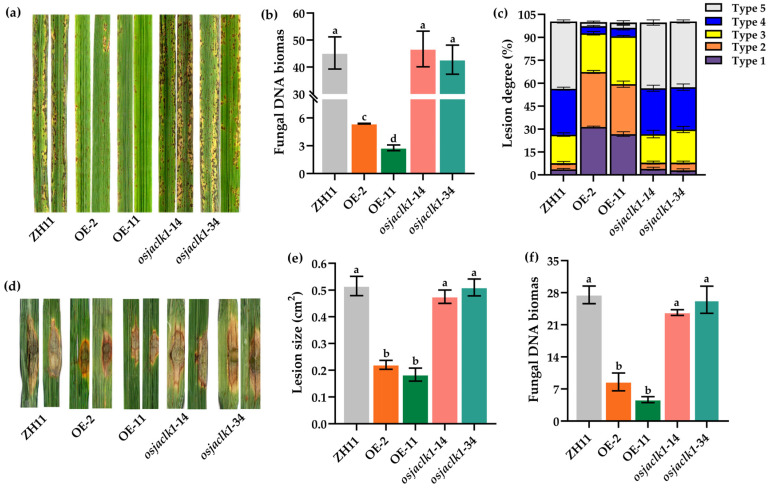
Overexpression of *OsJacLK1* in rice conferred resistance against *M. oryzae*. (**a**) Overexpression and gene deletion of the *OsJacLK1* rice plant were sprayed with rice blast fungus Guy11 after 7 d; (**b**) fungal biomass in infected leaves of rice mutants after spraying inoculation with Guy11. (**c**) Lesion degree on inoculated *OsJacLK1* mutants according to Rice Blast Resistance Comprehensive Index from International Rice Research Institute [36]. Level 1: small brown specks (<0.5 mm); Level 2: small spindle lesions (1–2 mm); Level 3: typical spindle lesions (2–3 mm); Level 4: larger lesions (3–5 mm); Level 5: coalescing lesions (>5 mm). (**d**) Punch inoculation onto *OsJacLK1* mutant leaves; (**e**) lesion size on infected leaves after punch inoculation; (**f**) fungus biomass in infected leaves after punch inoculation. Data are presented as the mean ± SD. Different alphabet letters above the columns indicate significant differences between groups (*p* < 0.05, one-way ANOVA followed by Tukey’s post hoc test).

To evaluate the capacity of resistance to rice blast disease, conidia spraying and punch inoculation were applied onto the leaves of *OsJacLK1* mutants (OE and gene-loss-function). In the spraying inoculation assay, seedlings of *OsJacLK1* overexpressed mutants OE-2 and OE-11 both exhibited much smaller lesion size (Figure 7a,b) and lower accumulation of fungal biomass (Figure 7c) in comparison with wild-type ZH11.

To evaluate the resistance of *OsJacLK1* to rice blast disease, we challenged *OsJacLK1* transgenic lines (overexpression, loss-of-function mutants) with *M. oryzae* using both conidial spray and punch inoculation methods. In the conidia spraying assay, overexpression mutant lines OE-2 and OE-11 exhibited significantly smaller lesion sizes (Figure 7a,b) and lower fungal biomass accumulation (Figure 7c) compared to wild-type ZH11 rice. In contrast, the gene-deletion mutants *(osjaclk1-14* and *osjaclk1-34*) displayed similar susceptibility to that of wild-type ZH11 rice. Consistent results were obtained from punch inoculation assays, where overexpression lines showed enhanced resistance and loss-of-function mutants exhibited similar susceptibility to the wild type (Figure 7d–f), further supporting that overexpression of *OsJacLK1* enhances rice resistance to rice blast fungus.

### 2.8. OsJacLK1 Triggered the Defense Response in Rice

**Figure 8:**
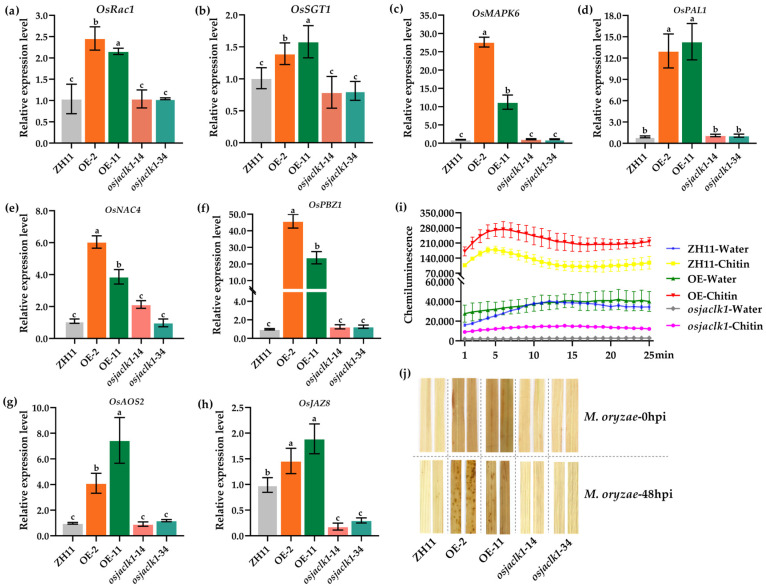
Overexpression of *OsJacLK1* triggers defense responses in rice. (**a**–**h**) Defense responsive genes *OsRac1*, *OsSGT1*, *OsMAPK6*, *OsPAL1*, *OsNAC4*, *OsPBZ1*, *OsAOS2* and *OsJZA8* in ZH11, *OsJacLK1* overexpressed rice (OE-2, OE-11), and gene-loss-function lines (*osjaclk1-14*, *osjaclk1-34)*, error bars represent the SD (*n* = 3). The alphabet letters above each column indicate significance. (**i**) Chitin-triggered ROS burst monitored in ZH11 and the rice mutants, error bars represent the SE (*n* = 3); (**j**) DAB staining of peroxide in rice lines mentioned above before and after *M. oryzae* spraying inoculation.

To elucidate the molecular basis of *OsJacLK1*-mediated immunity against *M. oryzae*, we examined the expression of defense-related genes in *OsJacLK1* mutant rice. Transcript levels of defense marker genes in pattern-triggered immunity (PTI) pathway [37,38,39,40,41,42,43,44,45], *OsRac1* (2.0- to 2.5-fold), *OsSGT1* (1.3- to 1.6-fold), *OsMAPK6* (8- to 27-fold), *OsPAL1* (10- to 80-fld), *OsNAC4* (4- to 6-fold), *OsPBZ1* (6- to 48-fold), *OsAOS2* (4- to 32-fold), and *OsJAZ8* (1.5- to 1.8-fold) were markedly activated in *OsJacLK1* overexpression lines, while their expression was significantly compromised in the gene knockout mutants (*osjaclk1-14*, *osjaclk1-34*), which showed no significant difference from the wild-type ZH11 rice (Figure 8a–h).

We next monitored ROS production in response to chitin elicitation. Chitin-triggered ROS burst was significantly enhanced in *OsJacLK1* overexpression lines (OE-2 and OE-11) relative to ZH11 (Figure 8i). Conversely, ROS accumulation was attenuated in the *osjaclk1-14* and *osjaclk1-34* mutants, remaining lower than that in chitin-treated ZH11 plants. Furthermore, 3,3′-Diaminobenzidine (DAB) staining revealed increased accumulation of hydrogen peroxide in the leaves of OE-2 and OE-1 before *M. oryzae* inoculation. At 48 h post-inoculation (hpi), a much stronger accumulation of peroxide was observed in the overexpressing mutants, while it was nearly absent in the gene knockout mutants (Figure 8j).

Collectively, these results demonstrate that overexpression of *OsJacLK1* triggers defense-related gene activation and ROS accumulation, thereby enhancing resistance to *M. oryzae*.

## 3. Discussion

Jacalin-related lectins have emerged as crucial regulators in plant stress responses, with functions ranging from direct antimicrobial activity in vitro to modulation of defense signaling, including pathogens, salt, drought, and temperature stresses [15]. In rice, the genetic functions of most jacalin-related lectins (26/30) remain uncharacterized, especially jacalin-like lectin kinases (JacLKs), which were specifically found in *Poaceae* so far [25,46], suggesting potential roles for environmental adaptation. In this study, we focused on a putative jacalin-related lectin-like kinase, *OsJacLK1*, and explored its role in mediating rice resistance to rice blast fungus. Our study indicates that *OsJacLK1* acts as a positive regulator of immune responses in rice and could serve as a potential candidate for enhancing disease resistance in rice breeding programs.

The structural organization of OsJacLK1 provides important insights into its potential mode of action. Besides the phosphate-anchoring motif, the kinase domain of OsJacLK1 contains HKN and PPE motifs instead of the canonical HRD and APE motifs (Figure 1b,c), indicating a lack of catalytic activity to transduce the signal by auto-phosphorylation, but retains a kinase-like fold [47]. This observation strongly suggests that OsJacLK1 may function as a putative “pseudokinase” or “kinase-like” protein. Pseudokinase-mediated signaling has emerged as an important regulatory mechanism in both animal and plant systems, wherein scaffold proteins coordinate the assembly of signaling complexes without contributing catalytic activity themselves [48,49,50,51,52]. This structural feature distinguishes OsJacLK1 from canonical RLKs and positions it as a potential signaling hub rather than an enzyme, enabling it to serve as a scaffold that recruits and facilitates phosphorylation events mediated by other active kinases, which require further investigation through kinase assays or protein interaction studies.

We further demonstrated that recombinant OsJacLK1 protein possesses both hemagglutination activity and carbohydrate-binding capacity, specifically interacting with mannose, chitin, and *M. oryzae* cell wall components (Figure 2a,b). These properties align with properties of canonical JRL, OsMBL1 [27,31], and suggest that OsJacLK1 may function as a pattern recognition receptor (PRR) or co-receptor capable of directly sensing pathogen-associated molecular patterns (PAMPs) such as chitin. The expression patterns of *OsJacLK1* support its role as a node for stress signal integration as well as other jacalin lectins, such as *OsMBL1* and *OsJAC1* [27,31,32,33,34,35]. The differential expression patterns of *OsJacLK1* in response to various phytohormones provide insight into its potential role in integrating distinct stress signaling pathways. The rapid and strong induction of *OsJacLK1* by both SA (20-fold at 6 h) and Me-JA (55- to 58-fold) (Figure 3a,b) is particularly notable, as these two hormones typically mediate antagonistic defense pathways against biotrophic and necrotrophic periods, respectively [53,54,55,56]. Although SA and JA signaling are often antagonistic, the fact that *OsJacLK1* was strongly up-regulated by both SA and MeJA suggests that it may be co-regulated by these two defense hormone pathways, which was verified by the transcriptional activation of SA pathway marker genes (including *OsPAL1*, *OsPBZ1*, and OsSGT1, etc.) and JA pathway marker genes (*OsAOS2* and *OsJAZ8*) in *OsJacLK1*-overexpressed rice (Figure 8).

The moderate but sustained up-regulation by ABA aligns with its role in abiotic stress responses [57] and is consistent with the moderate salt-induced expression of *OsJacLK1* (Figure 4). Additionally, emerging evidence indicates crosstalk between auxin and defense pathways, where auxin signaling can influence susceptibility or resistance depending on the pathogen [58,59]. The pronounced IAA response of OsJacLK1 raises the possibility that it may also participate in growth-defense trade-offs, potentially linking developmental cues to stress readiness. However, this hypothesis needs further verification, especially on agronomic traits and physiological properties of *OsJacLK1* mutants. Notably, the involvement of *OsJacLK1* in the early immune response against *M. oryzae* is further supported by its activation upon chitin oligosaccharide treatment (3.08-fold within 1.5 h; Figure 5a), a known PAMP recognized by plants to initiate defense response, leading to the activation of downstream immune responses, including the production of the expression of defense-related genes and ROS burst [60,61].

Overexpression of *OsJacLK1* in rice conferred enhanced resistance to *M. oryzae* (Figure 7), consistent with previous studies on jacalin-related lectins OsJAC1, OsMBL1 [24,27,31], which have been shown to positively modulate defense responses against rice blast fungus. The enhanced resistance observed in *OsJacLK1*-overexpressing rice plants was accompanied by elevated profiles of multiple defense-related genes (Figure 8a–h), stronger ROS bursts and H_2_O_2_ accumulation (Figure 8i,j).

In chitin-triggered PTI, OsRac1 functions as an early molecular switch downstream of the receptor complex, OsMAPK6 operates in a dedicated MAPK cascade that drives phytoalexin biosynthesis, OsSGT1 serves as a reliable transcriptional marker of PTI activation, and OsNAC4 acts as a downstream NAC transcription factor in this defense pathway [44,62,63]. All four genes were significantly up-regulated in *OsJacLK1* overexpressed plants, compared to the wild type. In the OsRac1-OsMAPK6 signaling cascade, the transcription factor RAI1 is activated downstream and directly regulates *OsPAL1* expression [64]. Consistent with this, our *OsJacLK1* overexpression lines showed up-regulation of *OsPAL1*, suggesting that *OsJacLK1* activates this core PTI pathway. Furthermore, the increased expression of *OsAOS2* and *OsPBZ1* likely reflects downstream activation of JA and SA branches, respectively, while *OsJAZ8* up-regulation may represent a feedback regulatory response within the JA pathway. This observation demonstrates that constitutive expression of *OsJacLK1* primes a core PTI signaling module OsRac1-OsMAPK6 cascade, leading to the transcriptional induction of downstream components such as *OsSGT1*, *OsNAC4* and SA-/JA synthesis genes.

Furthermore, the mechanism basis of *OsJacLK1*-mediated resistance also involves modulation of ROS dynamics. ROS production is one of the earliest responses to pathogen attack and plays a crucial role in limiting pathogen spread in two ways: as direct antimicrobial agents and as second messengers that activate downstream defense responses [60,61]. Under resting conditions (without chitin treatment or pathogen inoculation), *OsJacLK1* overexpression lines exhibited basal ROS and H_2_O_2_ levels comparable to those of wild-type plants. However, upon chitin oligosaccharide elicitation or *M. oryzae* inoculation, overexpression lines displayed markedly enhanced ROS bursts and elevated H_2_O_2_ accumulation, whereas knockout lines showed reduced ROS responses (Figure 8i,j). This pattern indicates that *OsJacLK1* primes the plant’s oxidative burst machinery rather than constitutively activating it—a hallmark of a potentiated PTI state. The enhanced ROS accumulation in *OsJacLK1*-overexpressing plants, coupled with the up-regulation of defense genes, likely contributes to the observed resistance phenotype through both mechanisms. By priming the ROS-generating system and defense gene expression, OsJacLK1 enables a faster and stronger response upon pathogen challenge, ultimately restricting fungal invasion and conferring enhanced resistance against *M. oryzae*. Presumably, this function might align with the proposed lectin-like pseudokinase hypothesis, wherein *OsJacLK1* might serve as a scaffold that senses chitin released from *M. oryzae* and facilitates the assembly and activation of oxidase complexes, or MAPK cascades that regulate ROS production. The reduced ROS burst in phenotypically unaltered knockouts suggests functional redundancy in maintaining basal resistance.

Additionally, there was no visible phenotype in *OsJacLK1* knockout lines upon *M. orzyae* inoculation. This phenomenon could be caused by several factors. The rice genome encodes 30 *JRL* genes with overlapping specificities [22], and *JRLs* from other plants are typically stress-inducible rather than constitutively expressed [22,65,66,67], which may explain the absence of a knockout phenotype under standard growth conditions. Furthermore, recent studies have shown that some JRLs act in specific cell types under ER stress and may be compensated by other chaperones [11,20]. Consistent with these observations, we found that overexpression or deletion of certain rice *JRLs* did not alter blast resistance. This is in line with reports that *JRL* overexpression effects are background-dependent. For instance, *OsJAC1* overexpression enhances resistance in some backgrounds (e.g., ZH10 or Dongjing) but not in ZH11, where it instead functions as a negative regulator [23,68]. These contrasting results suggest that the effect of a given JRL may depend on the specific gene, the genetic background, and the pathogen isolate used. Therefore, the absence of clear phenotypes in our lines may reflect functional redundancy, condition-dependency, or negative regulation.

Although *OsJacLK1* was modestly induced under salt stress, neither overexpression nor knockout lines displayed significant phenotypic differences from wild-type plants when subjected to salinity challenge, suggesting that *OsJacLK1* may be dispensable for salinity tolerance. However, given that *OsJacLK1* belongs to a large gene family, it remains possible that it participates in a redundant regulatory pathway in which its function is compensated for by other homologous genes. Further detailed investigation will be needed to address this possibility.

In summary, our study demonstrates that *OsJacLK1* is a positive regulator of rice immunity against *M. oryzae*. The enhanced resistance observed in *OsJacLK1*-overexpressing lines is associated with increased ROS production and up-regulation of defense-related genes, providing valuable insights into the molecular mechanisms of rice immunity. Despite its limited role in salinity tolerance, *OsJacLK1* holds promise as a candidate for genetic improvement of disease resistance in rice.

## 4. Materials and Methods

### 4.1. Bioinformatics Analysis of OsJacLK1

The protein domain composition was analyzed by using NCBI CD Search [69]. N-glycosylation sites were predicted with NetNGlyc 1.0 [70], and O-glycosylation sites were predicted using NetOGlyc 4.0 [71]. Protein phosphorylation sites were predicted using NetPhos 3.1 [72]. The protein structure prediction was performed with SWISS-MODEL [73,74].

Multiple sequence alignment for OsJacLK1 with active and inactive kinase sequences, was performed using PRALINE (https://www.ibi.vu.nl/programs/pralinewww/, accessed on 1 March 2026) with default settings [75]. Multiple sequence alignment for OsJacLK1 and its orthologs in plants was performed by using ClustalW with default parameters in MEGA12 (version 12.0; default parameters) [76]. The phylogenetic analysis, the tree was constructed by MEGA12 using a Maximum Likelihood method (with 1000 bootstrap replicates) and displayed by Interactive Tree of Life (iTOL) [77].

For phylogenetic analysis, involved sequences were obtained from the National Center for Biotechnology Information databases (https://www.ncbi.nlm.nih.gov/genbank/, accessed on 30 August 2025), the Rice Genome Annotation Project (https://rice.uga.edu/analyses_search_blast.shtml, accessed on 30 August 2025) and the Arabidopsis Information Resource (https://www.arabidopsis.org/, accessed on 30 August 2025).

### 4.2. Growth of Plants

Varieties of rice (*Oryza sativa*) were grown in a growth chamber at 28 °C during the daytime with 16 h of light and 8 h of darkness. The japonica Zhonghua11 (ZH11) seeds were used for transformation [78] to generate *OsJacLK1* gene-overexpressing rice mutants and gene-loss-function mutants.

### 4.3. Vector Construction and Transgenic Plant Validation

To generate *OsJacLK1* overexpression rice, the coding region of *OsJacLK1* was cloned into the plant binary vector pCXUN with a C-terminal enhanced-GFP tag under control of the maize ubiquitin promoter [79].

For gene knockout construction, two target sequences, 5′CTGCTATGACACAAGACGTA3′ and 5′ GAAATTTGACGTGTTC-AGTC3′ from the *OsJacLK1* exon region, were amplified and fused with U6a-sgRNA cassette and U6b-sgRNA cassette, respectively, which were subsequently constructed into CRISPR/Cas9-mediated genome editing vector pYLCRISPR/Cas9Pubi-H [80]. The recombinated plasmid vectors were then transformed into *Agrobacterium tumefaciens* EHA105. Genetic transformation was performed by Biorun Co., Ltd. (Wuhan, China).

### 4.4. Rice Blast Disease Inoculation

*M. oryzae* Guy11 strain was grown in starch yeast extract medium for hyphal growth at 28 °C. Conidia were produced by growing cultures on rice bran medium (4% [*w*/*v*] rice bran and 1.5% [*w*/*v*] agar, pH 6) for 5 d in the dark, followed by exposure to a 12 h light/dark cycle for a further 2 d at 25 °C [81].

To carry out plant inoculation assays, conidia were harvested from plate cultures and suspended at a concentration of 3 × 10^5^ spores/mL in 0.02% (*v*/*v*) Tween 20 water solution. For dynamic transcription profiles of *OsJacLK1* in response to rice blast fungus, the four-week-old ZH11 seedlings were sprayed with Guy11 spore suspension, and kept in dark and high-humidity conditions for 18 to 24 h. Seedlings sprayed with 0.02% Tween 20 in water served as the control. Leaves were harvested at different time points (0, 12, 24, 48, and 72 h, respectively) and RNA was extracted, reverse-transcribed, and *OsJacLK1* expression changes were analyzed by qRT-PCR. Rice *OsActin* was used as the internal reference gene. The relative expression levels were calculated using the 2^−ΔΔCt^ method [82]. To determine the sensitivity toward *M. oryzae*, the four-week-old seedlings of *OsJacLK1*-overexpressing mutants (OE-2, OE-11) and gene knockout mutants (*osjaclk1-14*, *osjaclk1-34*) were sprayed with Guy11 spore suspension. After being shielded from light for 18–24 h, the plants were then exposed to light with 16 h of light and 8 h of dark. Disease severity was assessed at 7 d after inoculation using the 1–5 scale of the International Rice Research Institute (IRRI) [36]. Level 1: small brown specks (<0.5 mm); Level 2: small spindle lesions (1–2 mm); Level 3: typical spindle lesions (2–3 mm); Level 4: larger lesions (3–5 mm); and Level 5: coalescing lesions (>5 mm), often with chlorosis and necrosis. Disease index (DI) was calculated as DI = [Σ (*n* × s)/(*N* × 5)] × 100, where *n* = number of leaves with score s, and *N* = total number of leaves. Each treatment consisted of three replicate pots with ten plants per pot, and experiments were repeated three times independently. For each replicate, a minimum of 20 leaves were randomly collected and examined for lesion development. Lesions with clear borders and typical blast symptoms were selected, avoiding leaf margins and midribs. Lesions per leaf were measured using ImageJ2.

For the punch inoculation disease assay, more than ten detached leaves from each rice line were punch-inoculated with *M. oryzae* by following a previous study [27]. The lesion size in each leaf was quantified using the software ImageJ2.

The relative fungal growth was evaluated by the DNA amount ratio of *M. oryzae MoPot2* to the rice *OsUbi* gene by using real-time PCR. Three independent repeats are performed in each assay.

### 4.5. Transcripts of OsJacLK1 to Exogenous Hormones, Chitin, Salt, and M. oryzae

According to the method presented by references [23,83,84], ten-day-old ZH11 plants were sprayed with 100 μM MeJA, 1 mM SA, 10 μM ABA, and 20 μM IAA solutions, respectively. Rice leaves were collected at different time points post-treatment (0, 3, 6, 12, 24, 36, and 48 hpi).

For chitin treatment, five-day-old sterilized ZH11 seedlings were submersed in a sterilized 500 μg/mL chitin oligosaccharide solution. The whole plants were collected at 1, 1.5, 3, and 6 h after immersion.

For the salt stress assay, NaCl was applied to the two-week-old ZH11 rice seedlings that grew hydroponically at a final concentration of 150 mM. Samples included with roots and leaves were harvested at 0, 12, 24, 48, 72, and 96 h after stress.

For *M. oryzae* inoculation, four-week-old ZH11 seedlings were sprayed with comparable strain Guy11 spores. The second extended leaf from each plant was collected at 0, 12, 24, 48, 72, and 96 h post-inoculation.

Rice samples under each treatment were harvested for RNA extraction and reverse transcription. The water-treated plants were set as a control for each treatment. Transcriptional profiles of the *OsJacLK1* gene were monitored by qRT-PCR (Mastercycler, Eppendorf) and normalized with water treatment at 0 h. *OsActin* was set as an internal reference. Each treatment was performed at least three biological repeats. The 2^−ΔΔCt^ method was used for relative quantification.

### 4.6. Expression of Defense-Responsive Genes in Transgenic Rice

qRT-PCR was used to analyze the expression changes in defense-related genes *OsRac1*, *OsSGT1*, *OsPBZ1*, *OsPAL1*, *OsAOS2*, *OsMAPK6*, *OsAOS2*, and *OsJAZ8* in the leaves of wild-type ZH11 and *OsJacLK1* transgenic rice plants, including overexpression line OE-2, OE-11, and gene deletion mutants *osjaclk14* and *osjaclk-34*. The actin gene *OsActin* was set as an internal reference. Gene expression of each defense-related gene was normalized with that in wild-type ZH11.

### 4.7. ROS Burst Measurement and DAB Staining

Measurement of ROS burst in rice leaf disks was performed as described [27] with modifications. Leaf disks from four-week-old rice seedlings were cut with a mouse ear punch and pre-incubated in sterile distilled water overnight. Three leaf disks for each sample were placed on a 96-well ELISA PLATE containing 10 mM luminol, 5 mg/mL horseradish peroxidase, and 8 nM (GlcNAC)_7_, or water as a control. After treatment, ROS production in rice tissues was immediately monitored by luminescence assay with Thermo Scientific Varioskan Flash Microplate Readers (Waltham, MA, USA). Three replications were performed for each sample and treatment. SE values were calculated for each treatment.

For DAB staining, the *M. oryzae* inoculated leaves from each line at 48 hpi were harvested and submerged in 1 mg/mL of DAB solution (pH 3.8). After vacuum infiltration (~0.05–0.08 MPa) for about 15 min, the leaf samples were incubated in DAB solution for another 8 h in darkness. Then transfer the leaves to 95% ethanol, and incubate in a water bath at 90 °C for 20–30 min until green chlorophyll is extracted and the background becomes pale.

## Data Availability

The original contributions presented in the study are included in the article; further inquiries can be directed to the corresponding authors.

## Supplementary Materials

The following supporting information can be downloaded at: https://www.mdpi.com/article/10.3390/plants15091376/s1. Figure S1: multiple sequence alignment of OsJacLK1 and homologous proteins in plants; Figure S2: tissue-specific expression of *OsJacLK1* in rice seedlings. Table S1: primers used in this study.
