# Supplementary material for: Jacalin-Related Lectin OsJacLK1 Positively Regulates Resistance to Magnaporthe oryzae in Rice
Source: Plants (Basel). 2026 Apr 30;15(9):1376. doi: 10.3390/plants15091376 (PMC13165441; doi:10.3390/plants15091376)
Supplement: Supplementary file 1 [file plants-15-01376-s001.zip › Supplemental data.pdf]

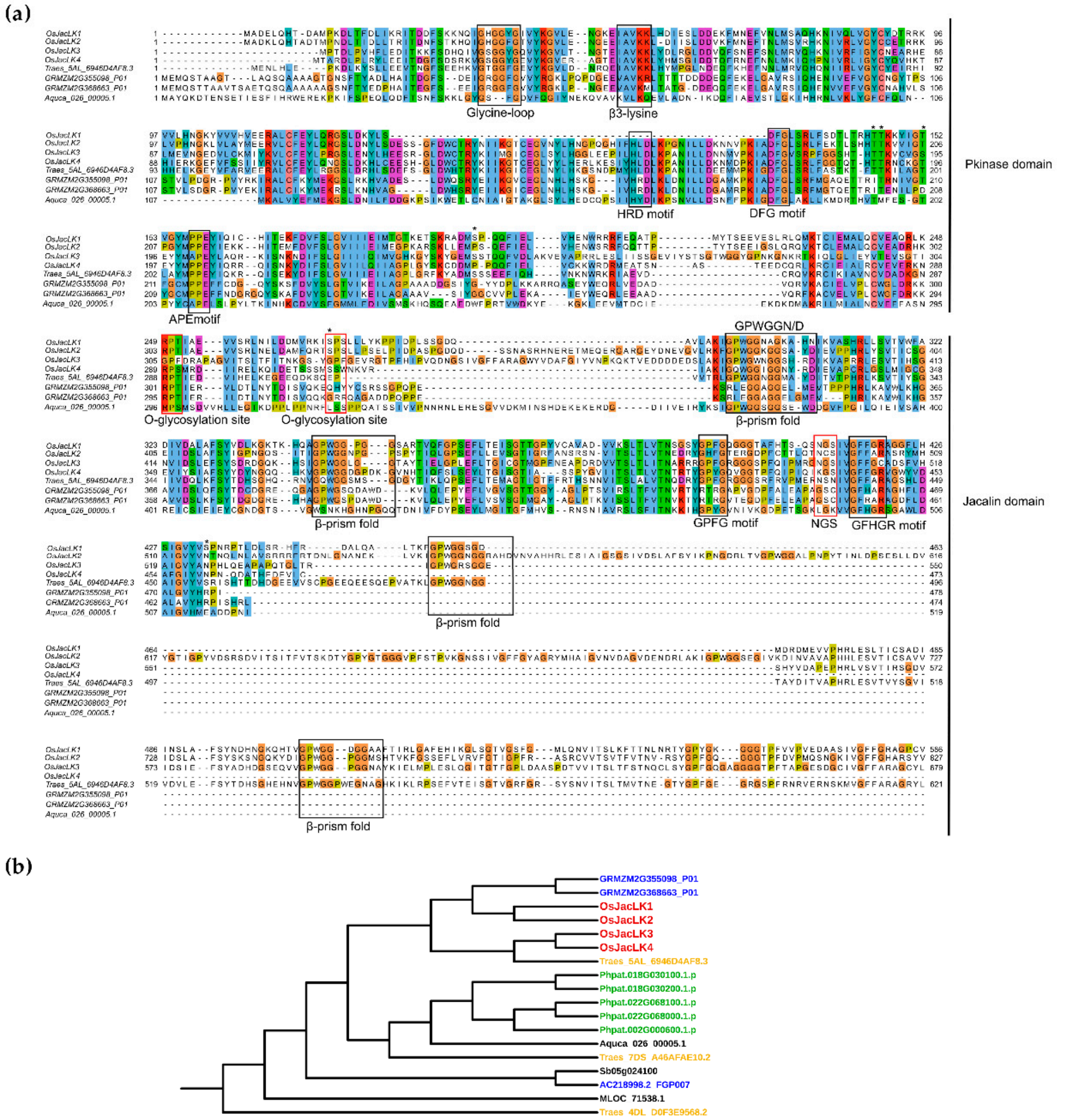

Figure S1 Sequence alignment and phylogenic analysis of OsJacLK1 with its homologs in plants. (a) Sequence alignment of OsJacLK1 and its homologous proteins. Motifs were labeled with rectangles in dark. Glycosylation sites were marked with rectangles in red.(b) Phylogenic analysis of OsJacLK1 with its homologs in maize, wheat, barley, sorghum, rocky mountain columbine (*Aquilegia coerulea*, Aquca_026_00005, spreading earthmoss (*Physcomitrium patens*: Phpat.022G000600.1, Phpat.022G068000.1, Phpat.022G068000.1, Phpat.018G0300100.1, Phpat.018G0300200.1).

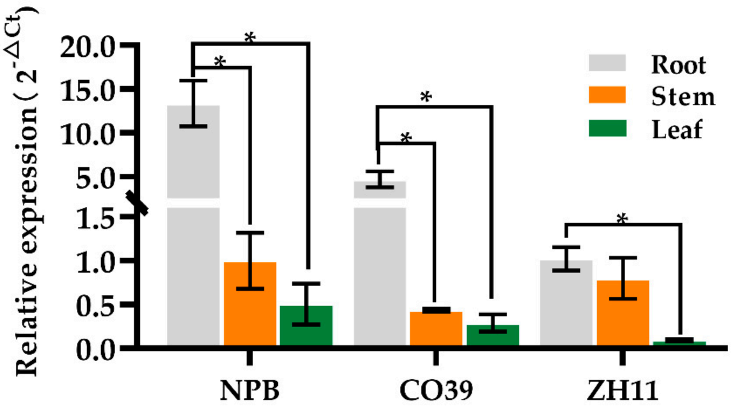

Figure S2 Tissue specific expression of *OsJacLK1* in rice seedlings. Roots, stem and leaves were harvested from different rice cultivar, including Nipponbare(NPB), CO39, and Zhonghua 11(ZH11), at four-week-old stage. Total RNA was extracted and subjected to qRT-PCR analysis. Expression levels were normalized to an internal control *OsActin.* Values represent means ± SD from three independent biological replicates.

Table S1 Primers used in this study

Primers	Sequence (5' to 3')	Description
OsJacLK1-OE-F	GTTGTTTGGTGTTACTTCTGCAGCCCGGGAT GGCGGACGAGTTACAACACAC	Overexpression vector construction
OsJacLK1-OE-R	CTCCTCGCCCTTGCTCACCATGGATCCCAA GTATGTGCGAATATAGACCCCA	
OsRac1-QF	GTGTTCATCCTGTCCTTCTCC	Real-time PCR
OsRac1-QF	AGGCCCTATCTTCACGGAG	
OsSGT1-QF	TCATCCCTGAGAAAAGCAGATAC	Real-time PCR
OsSGT1-QR	AAGGATATGATGGCCTCTGG	
OsMAPK6-QF	AGTCTGCTCATCACCCTTCAG	Real-time PCR
OsMAPK6-QR	ACCTTCAGCTTTGAACCAGGG	
OsNAC4-QF	TCCTGCCACCATTCTGAGATG	
OsNAC4-QR	TTGCAGAATCATGCTTGCCAG	
OsPAL1-QF	AGGAGCTCGGCTGCGTATT	Real-time PCR
OsPAL1-QR	ATGCCGAGGAACACCTTGTT	
OsPBZ1-QF	CCCTGCCGAATACGCCTAA	Real-time PCR
OsAOS2-QF	CAATACGTGTACTGGTCGAATGG	
OsAOS2-QR	AAGGTGTCGTACCGGAGGAA	
OsJAZ8-QF	GAAAGTGCAAGTGAGGCAGC	Real-time PCR
OsJAZ8-QR	ATCCTTGACCTTGGTGGACG	
OsActin-QF	ACACCAACAATCCCAAACAGAG	Real-time PCR
OsActin-QR	GAGTATGATGAGTCGGGTCCAG	
OsJacLK1-QF	ACACTTCGCAGAGCAATGGA	Real-time PCR
OsJacLK1-QR	TGAGAAGGCAAGCGAGTTGA	
MoPot2 -QF	ACGACCCGTCTTTACTTATTTGG	Real-time PCR
MoPot2-QR	AAGTAGCGTTGGTTTTGTTGGAT	
Ubi-QF	GCCCAAGAAGAAGATCAAGAAC	Real-time PCR
Ubi-QR	AGATAACAACGGAAGCATAAAAGTC	
