# Supplementary figures and images for: Jacalin-Related Lectin OsJacLK1 Positively Regulates Resistance to Magnaporthe oryzae in Rice
Source: Plants (Basel). 2026 Apr 30;15(9):1376. doi: 10.3390/plants15091376 (PMC13165441; doi:10.3390/plants15091376)

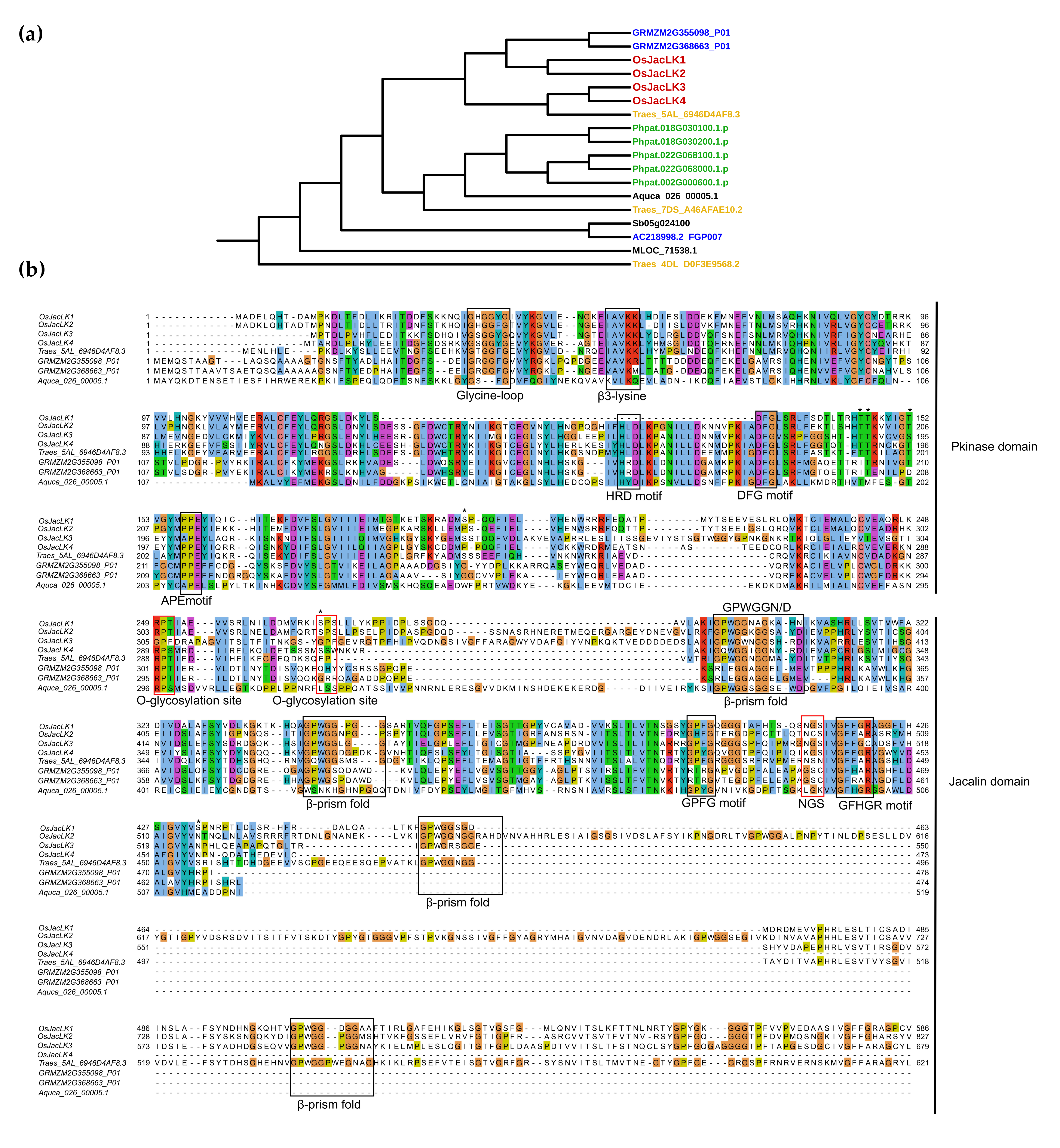

Supplement: Supplementary file 1 [file plants-15-01376-s001.zip › Figure S1.tif]

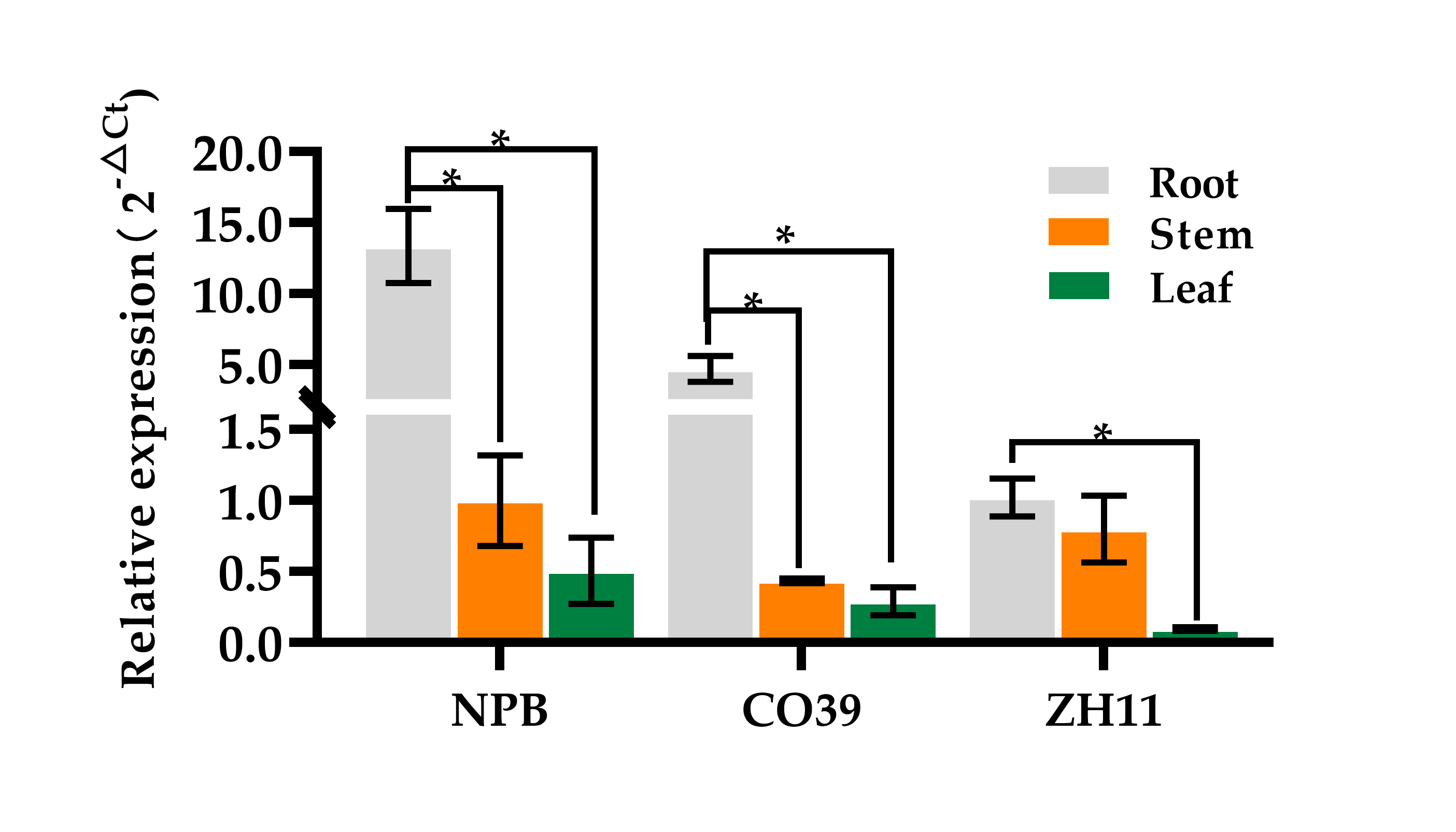

Supplement: Supplementary file 1 [file plants-15-01376-s001.zip › Figure S2.tif]
